# Feasibility of filamentous fungi for biofuel production using hydrolysate from dilute sulfuric acid pretreatment of wheat straw

**DOI:** 10.1186/1754-6834-5-50

**Published:** 2012-07-23

**Authors:** Yubin Zheng, Xiaochen Yu, Jijiao Zeng, Shulin Chen

**Affiliations:** 1 Department of Biological Systems Engineering, L.J. Smith Hall, Washington State University, Pullman, WA, 99164-6120, USA

**Keywords:** Filamentous fungi, *Mortierella isabellina*, Microbial lipid, Biodiesel, Lignocellulosic biomass, Wheat straw

## Abstract

**Background:**

Lipids produced from filamentous fungi show great promise for biofuel production, but a major limiting factor is the high production cost attributed to feedstock. Lignocellulosic biomass is a suitable feedstock for biofuel production due to its abundance and low value. However, very limited study has been performed on lipid production by culturing oleaginous fungi with lignocellulosic materials. Thus, identification of filamentous fungal strains capable of utilizing lignocellulosic hydrolysates for lipid accumulation is critical to improve the process and reduce the production cost.

**Results:**

The growth performances of eleven filamentous fungi were investigated when cultured on glucose and xylose. Their dry cell weights, lipid contents and fatty acid profiles were determined. Six fungal strains with high lipid contents were selected to culture with the hydrolysate from dilute sulfuric acid pretreatment of wheat straw. The results showed that all the selected fungal strains were able to grow on both detoxified liquid hydrolysate (DLH) and non-detoxified liquid hydrolysate (NDLH). The highest lipid content of 39.4% was obtained by *Mortierella isabellina* on NDLH. In addition, NDLH with some precipitate could help *M. isabellina* form pellets with an average diameter of 0.11 mm.

**Conclusion:**

This study demonstrated the possibility of fungal lipid production from lignocellulosic biomass. *M. isabellina* was the best lipid producer grown on lignocellulosic hydrolysates among the tested filamentous fungi, because it could not only accumulate oils with a high content by directly utilizing NDLH to simplify the fermentation process, but also form proper pellets to benefit the downstream harvesting. Considering the yield and cost, fungal lipids from lignocellulosic biomass are promising alternative sources for biodiesel production.

## Background

The traditional feedstocks for biodiesel production are vegetable oils and animal fats resulting in competition with the food industry. Single cell oil (SCO) from microbes is considered as an alternative oil source due to the high productivity and low land requirement [1]. Among different oleaginous microorganisms, increasing attention has been paid to filamentous fungi due to multiple advantages: (1) Accumulate up to 80% of lipid and produce some value-added fatty acids [2]. Aggelis [3] cultured *Cunninghamella echinulata* to achieve 46.6% of cellular lipid with a γ-linolenic acid (GLA) content of 14.1%. Moreover, it was demonstrated that the arachidonic acid (AA) content in *Mortierella alpine* was more than 16% of dry cell weight and the total lipid also reached 36% [4]. (2) Show good lipid profiles for making high quality biodiesel. Vicente et al. [5] suggested that not all lipids extracted from microbes were suitable for biodiesel production but only saponifiable lipids and free fatty acids could be produced to fatty acid methyl esters (FAMEs). Their results showed that 98.0% of the total lipids extracted from *Mucor circinelloides* were saponifiable lipids and free fatty acids, and the fungus-derived biodiesel met the specifications of the current existing standards very well; (3) Use a variety of carbon sources for lipid production, such as monosugar, glycerol, acetic acid, cereal, corncob, sweet sorghum, wheat straw, orange peel, apple pomace and oil [3,6-13]; (4) Produce oils through solid state fermentation with low capital cost and low energy expenditure [8]; (5) Tend to form pellets that not only reduce the viscosity of the fermentation broth to improve the mixing and mass transfer performance, but also are much easier to be harvested from cell broth by using simple filtration, compared with traditional high cost centrifugation methods [14].

Although SCO from filamentous fungi shows the promise for biodiesel production, the hurdle is the high production cost. It has been reported that up to 75% of the total costs came from the feedstocks or carbon sources required for producing microbial lipids [2]. However, the cost will be reduced potentially if cheap feedstocks or waste materials can be used. Xue et al. [15] successfully grew the oleaginous yeast *Rhodotorula glutinis* with monosodium glutamate wastewater to produce 25 g L^-1^ biomass with 25% lipid content. André et al. [6] reported that the fungus *Aspergillus niger* could accumulate 41–57% of lipid on biodiesel derived waste glycerol. Moreover, food wastes have proven to be suitable substrates for production of lipid by yeast and microalgae [16,17]. However, the availability of these sources is limited and not able to meet the increasing demand of alternative energy. It is very urgent, therefore, to investigate other renewable sources as feedstocks for microbial lipid production.

Lignocellulosic materials have attracted a lot of attention as feedstocks for biofuel production due to its abundance and relatively low cost. It was estimated that there would be potentially over 1.3 billion dry tons of lignocellulosic biomass produced in the US each year on a sustainable basis for biofuel production [18]. The energy content of this amount of biomass is equivalent to 3.8 billion barrels of oil, which is approximately more than half of the US’s annual energy consumption [19]. These inexpensive materials such as agricultural residues can result in a reasonable biofuel production cost [20]. Some studies have been conducted to produce lipid from oleaginous yeast by feeding with lignocellulosic material. Huang et al. [21] obtained a cell density of 28.6 g L^-1^ with 40% lipid content by culturing the yeast *Trichosporon fermentans* with detoxified rice straw hydrolysate. Yu et al. [22] reported that the yeast *Cryptococcus curvatus* could grow with non-detoxified wheat straw hydrolysate and reach 17.2 g L^-1^ dry cell weight with 33.5% lipid content. However, cultivation of filamentous fungi for lipid production with lignocellulosic hydrolysate has not been well examined.

The purpose of this study is to investigate the feasibility of culturing the filamentous fungi with lignocellulosic materials and to screen the best lipid producing strain, especially using the non-detoxified hydrolysates. The very basic requirements for fungi to be used for this purpose are: (1) can use various sugars, especially xylose; (2) can adapt to the lignocellulosic biomass processing without extensively conditioning the sugar stream; (3) can accumulate high lipid contents while utilizing lignocellulosics as the carbon source; (4) can grow with proper morphology to facilitate downstream processing.To achieve these objectives, the lipid accumulation capability of eleven filamentous fungal strains was evaluated on glucose and xylose respectively. Then, the selected strains with high lipid contents were cultivated with hydrolysates from dilute sulfuric acid pretreated wheat straw. The biomass and lipid yields, fatty acid profiles, capability to tolerate inhibitors and pellet formation were studied. Finally, the fungal lipid based biodiesel yield and cost were estimated when lignocellulosic biomass was used as the feedstock.

## Results and discussion

### Screening oleaginous fungi with xylose assimilation capability

Since xylose is the dominant component of the hydrolysate from the decomposition of hemicellulose during dilute sulfuric acid pretreatment, evaluation of lipid production by feeding oleaginous fungi with xylose is quite significant. Eleven potential lipid producing fungi, including *Aspergillus niger, Aspergillus terreus, Chaetomium globosum, Cunninghamella elegans, Mortierella isabellina, Mortierella vinacea, Mucor circinelloides, Neosartorya fischeri, Rhizopus oryzae, Mucor plumbeus, Thermomyces lanuginosus*, were cultured in the medium with xylose as the sole carbon source, while glucose was used in the control experiments. The fungal biomass and lipid productions are shown in Table 1. The results demonstrated that all the tested fungi could utilize xylose for their growth. *A. terreus* and *M. vinacea* showed the highest biomass production on xylose by reaching the dry cell weight (DCW) of 7.0 g L^-1^ and 7.1 g L^-1^ respectively. The lipid contents varied among different strains and ranged from 4.1% to 67.0% of DCW. There were six strains with the lipid contents above 20% on both glucose and xylose. The highest lipid contents were achieved by *M. isabellina* with 67.0% on glucose and 50.9% on xylose. Ratledge [23] stated that *N. fischeri* and *C. globosum* could accumulate more than 50% lipid, but our results showed the lipid contents were below 10% for these two strains. Also, *A. niger* poorly produced lipids on both glucose and xylose medium, although 41% lipid content was reported by feeding with glycerol [6]. 

**Table 1:** Fungal biomass and lipid production on glucose and xylose

**Strain**	**Substrate**	**Sugar consumed**	**DCW**	**Lipid content**	**Lipid concentration**	**Lipid yield**^**a**^
		**(g L**^**-1**^**)**	**(g L**^**-1**^**)**	**(%)**	**(g L**^**-1**^**)**	**(mg g**^**-1**^**)**
A. niger	Glucose	26.7	5.8	9.6	0.55	21
	Xylose	25.0	4.6	8.0	0.37	15
A. terreus	Glucose	24.8	7.2	37.4	2.70	109
	Xylose	27.8	7.0	31.9	2.22	80
C. elegans	Glucose	26.1	6.1	33.6	2.04	78
	Xylose	18.9	4.2	31.2	1.31	69
C. globosum	Glucose	9.5	2.3	5.1	0.13	14
	Xylose	13.4	3.3	4.1	0.15	11
M. circinelloides	Glucose	16.6	4.0	23.8	0.95	57
	Xylose	17.3	3.6	17.3	0.63	36
M. isabellina	Glucose	25.0	7.3	67.0	4.88	195
	Xylose	20.8	5.0	50.9	2.52	121
M. plumbeus	Glucose	25.8	4.8	20.6	0.99	38
	Xylose	27.7	5.9	16.6	0.97	35
M. vinacea	Glucose	25.4	7.3	51.9	3.79	149
	Xylose	26.7	7.1	43.9	3.12	117
N. fischeri	Glucose	17.5	5.9	8.8	0.52	30
	Xylose	17.1	5.4	8.9	0.48	28
R. oryzae	Glucose	23.4	3.2	34.8	1.09	47
	Xylose	18.3	3.3	20.0	0.66	36
T. lanuginosus	Glucose	18.7	5.8	21.0	1.22	65
	Xylose	16.7	4.6	20.4	0.93	56

^a^ Lipid yield: lipid concentration per gram sugar consumed.

**Figure 1:**
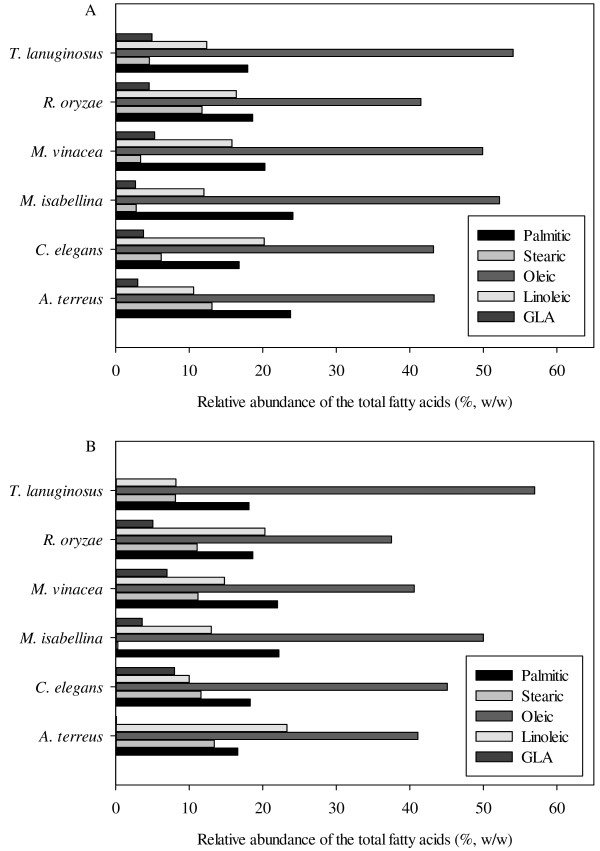
The major fatty acid profiles of six lipid producing fungal strains when cultured with (A) glucose and (B) xylose.

The fatty acid profiles are shown in Figure 1. The length of the fatty acid carbon chain ranged from 14 to 24 for these fungal strains (data not shown), and the major fatty acids were palmitic (C16:0), stearic (C18:0), oleic (C18:1) and linoleic (C18:2) and γ-linolenic (C18:3γ, GLA) acids. Oleic acid was the dominant fatty acid with the percentage of 40–60% of the total fatty acids for all the strains on both glucose and xylose. Significant differences were observed on the fatty acid compositions for different strains and substrates. Taking stearic acid for example, the lowest content was only 0.3% for *M. isabellina*, while *A. terreus* had the highest content of 13.4%. *M. vinacea* produced 3.4% and 11.2% of stearic acids on glucose and xylose respectively. Moreover, all the strains showed the capability to produce GLA on glucose, but xylose was not a suitable substrate for GLA accumulation by *A. terreus* and *T. lanuginosus*. The highest GLA content of 8% was achieved by *C. elegans*.

As one of the most abundant carbohydrates in nature, xylose can be easily released from biomass by hydrolysis, which makes it a potential feedstock for biofuel production [24]. However, compared with glucose (a more preferable substrate for most heterotrophic microbes), specific metabolic pathways are required for xylose utilization. Actually many microorganisms do not naturally use xylose as a substrate due to the lack of some key enzymes [25]. For instance, the most commonly used yeast *Saccharomyces cerevisiae* for ethanol production cannot ferment xylose naturally, which limits its industrial application. Therefore, the capability to utilize xylose to accumulate lipids is a critical criterion for screening strains with industrial potential in biodiesel area. In this study, all the eleven fungi candidates showed satisfactory results on xylose assimilation and more than half of them exhibited comparable or even higher biomass production on xylose than on glucose. Particularly, *A. terreus C. elegans M. isabellina M. vinaceaR. oryzae* and *T. lanuginosus* could accumulate more than 20% lipid on xylose (Table 1), which were selected for the following experiments utilizing wheat straw hydrolysate as the substrate.

### Chemical compositions of hydrolysates

**Figure 2:**
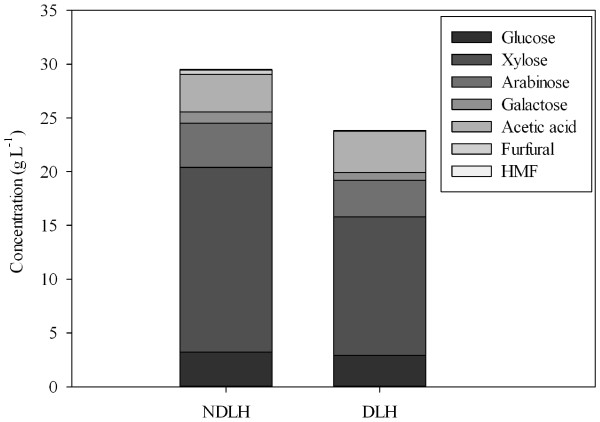
**Chemical compositions of NDLH and DLH.** Wheat straw was mixed with dilute sulfuric acid 2% (v/v) at a solid loading of 10% (w/v). The mixture was treated in an autoclave at 121°C for 60 min and the supernatant was separated for the analysis.

The chemical compositions of the non-detoxified liquid hydrolysate (NDLH) obtained after dilute sulfuric acid pretreatment of wheat straw are presented in Figure 2. Sugar monomers were the primary component in NDLH, which contained mainly glucose, xylose, arabinose and galactose. Xylose showed the highest concentration at 17.2 g L^-1^ which was 67.1% of the total sugars. Besides sugars, some other compounds were detected in NDLH, including 3.50 g L^-1^ acetic acid (from acetylation of hemicellulose), 0.39 g L^-1^ furfural and 0.04 g L^-1^ hydroxymethylfurfural (HMF) (from degradation of pentoses and hexoses). Acetic acid was generally considered as an inhibitor to ethanol producing organisms [26,27], however, as an organic acid, it can be used as the carbon source to support the growth of oleaginous yeast [1]. Furfural and HMF are also very toxic to many bacteria and yeasts [27,28]. In the overliming process, 92.8% of furfural and 58.0% of HMF were removed although most of acetic acid still remained in the detoxified liquid hydrolysate (DLH). On the other hand, the detoxification led to a loss of 22.2% sugars, which was in accordance with results reported by Yu et al. [22]. 

### Culture oleaginous fungi with NDLH and DLH

The selected six fungal strains were cultured using the hydrolysates from wheat straw pretreatment. The biomass, lipid contents, lipid concentrations and lipid yields are shown in Table 2. The results indicated that all the strains were able to grow on both NDLH and DLH. Although NDLH contained some inhibitors, such as acetic acid, furfural and HMF, all the tested strains except *C. elegans* and *T. lanuginosus* had a higher lipid concentration on NDLH than on DLH. Similarly, Yu et al. [22] demonstrated some oleaginous yeast strains also showed a higher lipid production on NDLH. The reason might attribute to not only the higher sugar concentration in NDLH, but also the inherent capabilities of these fungal strains to tolerate a certain amount of the toxic compounds. The highest DCW was achieved by *A. terreus* on NDLH but its lipid content was only 20.0%. *M. isabellina* and *M. vinacea* showed outstanding potential for lipid production since their lipid concentration exceeded 2 g L^-1^ on both NDLH and DLH. These two fungi demonstrated much higher lipid concentration than other tested strains and their lipid yields were at the same level as those in the cultures feeding with pure xylose (Table 1). 

**Table 2:** Culture of the selected fungal strains with NDLH and DLH

**Strain**	**Substrate**	**DCW**	**Lipid content**	**Lipid concentration**	**Lipid yield**
		**(g L**^**-1**^**)**	**(%)**	**(g L**^**-1**^**)**	**(mg g**^**-1**^**)**
*A. terreus*	NDLH	7.6	20.0	1.52	67
	DLH	7.3	17.6	1.28	69
*C. elegans*	NDLH	4.7	17.0	0.80	38
	DLH	3.8	23.1	0.88	50
*M. isabellina*	NDLH	6.7	39.4	2.63	117
	DLH	5.9	38.9	2.29	123
*M. vinacea*	NDLH	7.5	32.7	2.46	105
	DLH	7.0	29.7	2.07	110
*R. oryzae*	NDLH	5.2	16.1	0.84	38
	DLH	3.9	19.7	0.77	45
*T. lanuginosus*	NDLH	3.8	20.5	0.78	41
	DLH	4.3	21.3	0.92	51

Interestingly, higher lipid yields were obtained on DLH compared with NDLH for all the selected fungal strains. These results revealed that DLH was a more suitable substrate for these strains on sugar conversion to lipids, and the reason might ascribe to the negative impacts of the inhibitors in NDLH. However, as shown in Table 3, different treatments of the hydrolysate did not have significant impacts on the fatty acid profiles for all the strains except *A. terreus*. These were in accordance with the results reported by Hu et al. [29] and Yu et al. [22], who suggested that toxic compounds had a certain degree of inhibition on lipid content but did not affect the fatty acid profiles. For *A. terreus*, there were noticeable changes on the distributions of stearic acid, oleic acid and linoleic acid, and NDLH resulted in a lower polyunsaturated fatty acid (PUFA) content, which indicated *A. terreus* was more sensitive to inhibitors on the biosynthesis of fatty acids. All the selected fungi showed a high content of unsaturated fatty acid which would contribute to excellent fuel properties at low temperatures. However, excess PUFA will lead to lower oxidative stability. The European biodiesel standard (EN14214) specifies the iodine value lower than 120, cetane number higher than 51.0, viscosity in the range of 3.5–5.0 mm^2^ s^-1^, density in the range of 860–900 kg m^-3^, and requires the PUFA (> = 4 double bonds) and linolenic acid contents up to 1% and 12% respectively. The estimated biodiesel properties shown in Table 3 were calculated according to Ramírez-Verduzco et al. [30], and the results demonstrated that the fungal lipid matched the criteria very well, except *C. elegans* with more than 12% of GLA. In fact, the GLA can be separated independently due to its high value in the pharmaceutical areas [7], which cannot only improve the biodiesel quality but also reduce the overall production cost by creating an extra form of revenue. In summary, the filamentous fungi proved to be an ideal feedstock for biodiesel production since it had appropriate fatty acid profiles, and the estimated biodiesel properties from the fungal biomass met the requirements of existing standards [31]. 

**Table 3:** Fatty acid compositions of selected lipid producing fungal strains grown on NDLH and DLH

		**Relative abundance of the total fatty acids (%, w/w)**											
		***A. terreus***		***C. elegans***		***M. isabellina***		***M. vinacea***		***R. oryzae***		***T. lanuginosus***	
		**ND**^**a**^	**D**^**a**^	**ND**	**D**	**ND**	**D**	**ND**	**D**	**ND**	**D**	**ND**	**D**
Myristic	C14:0	0.3	0.2	0.4	0.6	0.7	0.9	0.4	0.5	0.2	0.2	0.3	0.4
Palmitic	C16:0	17.4	17.5	19.8	20.3	24.3	24.5	20.2	23.4	17.8	16.2	16.0	17.7
Palmitoleic	C16:1	0.6	0.4	1.3	0.8	2.6	1.4	2.3	1.8	0.5	0.8	0.2	1.0
Stearic	C18:0	8.5	12.5	9.2	6.2	3.8	5.6	2.8	3.6	16.0	11.8	9.4	9.9
Oleic	C18:1	57.0	42.3	41.6	34.8	47.8	52.4	53.3	51.3	35.4	40.5	60.3	56.5
Linoleic	C18:2	8.2	23.1	11.2	17.2	14.9	9.5	14.3	13.8	18.3	19.0	7.6	8.0
Linolenic	C18:3	0.6	0.1	11.3	12.4	2.0	2.3	3.7	2.2	4.8	5.5	0.7	1.0
Arachidic	C20:0	0.7	0.5	0.5	0.5	0.9	0.6	0.5	0.8	0.9	0.7	0.6	0.5
Lignoceric	C24:0	2.0	0.0	1.9	2.0	0.5	0.6	0.4	0.0	1.9	1.6	1.8	0.0
Unsaturated		70.2	68.6	67.1	70.4	69.2	67.2	75.4	71.6	61.7	68.1	71.3	70.8
PUFA		9.1	23.4	22.8	30.5	17.1	12.0	18.2	16.4	23.7	24.9	8.7	9.1
PUFA (> = 4 double bonds)		0.00	0.00	0.31	0.37	0.03	0.02	0.12	0.16	0.06	0.07	0.00	0.00
Iodine value^b^ (g of I_2_/100 g)		68.3	80.5	89.8	97.1	78.0	72.0	86.2	78.9	78.6	86.6	84.1	78.1
Cetane number^b^		61.4	59.4	57.2	54.8	59.4	60.9	57.8	59.4	59.7	57.9	61.5	61.6
Viscosity (mm^2^ s^-1^)^b^		4.5	4.5	4.3	4.2	4.4	4.4	4.3	4.4	4.5	4.4	4.5	4.5
Density (kg m^-3^)^b^		873	874	876	877	874	873	875	874	875	875	873	873
Higher heating value (MJ kg^-1^)^b^		39.8	39.8	39.7	39.7	39.7	39.8	39.8	39.8	39.8	39.8	39.8	39.8

^a^ ND means NDLH, D means DLH.

^b^ The iodine value, cetane number, viscosity, density and higher heating value were calculated according to Ramírez-Verduzco et al. [30].

**Figure 3:**
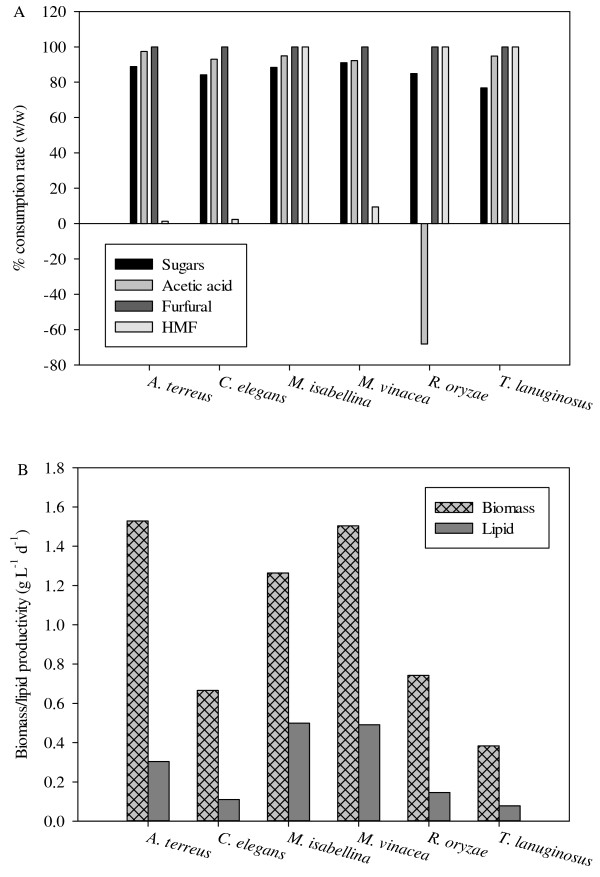
Culture of the selected fungal strains with NDLH: (A) Consumption rate of total sugars, acetic acid, furfural and HMF; (B) DCW and lipid productivity.

Consumptions of total sugars, acetic acid, furfural and HMF after the culture of fungi with NDLH are shown in Figure 3A. Furfural was completely depleted, but the consumption of HMF varied with different strains. Similar results showed that furfural was completely consumed by the yeast *C. curvatus* but HMF remained unconsumed during fermentation [22]. Furfural was considered as a more toxic compound than HMF for oleaginous yeast but could be reduced to a less inhibitory form furfuryl alcohol by furfural reductase [29]. The consumption of furfural indicated that the related metabolic pathways might exist in these filamentous fungi and furfural reductase was expressed when cultured with NDLH. However, NAD(P)H was used as the electron donor in this reduction, which made the furfural act as a redox sink, oxidizing NAD(P)H formed in biosynthesis [32]. This might be the reason why a lower lipid yield was observed when feeding with NDLH, since lots of reducing powers were required during the lipid synthesis. It has been reported that acetic acid was a very suitable substrate for lipid accumulation by oleaginous microbes [1,29]. Our data demonstrated that more than 90% of acetic acid was utilized by the selected fungal strains except *R. oryzae*. The consumption rate of *R. oryzae* was about −68%, which indicated that *R. oryzae* produced extra acetic acid into the culture medium. Karimi et al. [33] also reported that *R. oryzae* could produce acetic acid when fed with cellulosic substrates. Approximately 90% of the sugar consumption rate was obtained from the tested fungi except *C. elegans* and *T. lanuginosus*. The highest DCW productivity of 1.5 g L^-1^ d^-1^ was achieved by *A. terreus* and *M. vinacea* (Figure 3B). However, the best lipid producer was *M. isabellina* with a lipid content of 39.4% of DCW and lipid productivity of 0.5 g L^-1^ d^-1^ by feeding with NDLH. The capability to produce lipids using wheat straw hydrolysate without the detoxification process is very critical because it can simplify the fermentation process and potentially reduce the production cost. 

### Fungal pelletization when feeding with NDLH

**Figure 4:**
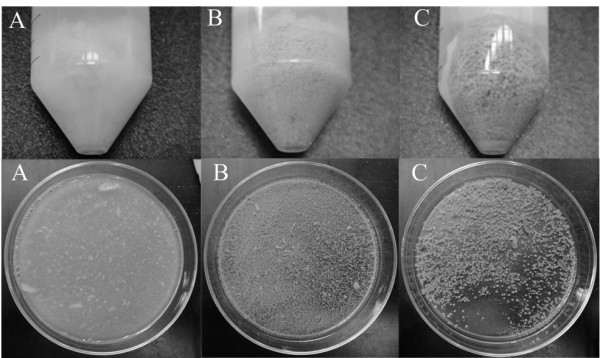
**Pelletization of *M. isabellina* with different culture medium: (A) basic medium supplemented with 30 g L**^**-1**^**xylose; (B) NDLH filtered with a 0.22 μm membrane after pH adjustment; (C) NDLH without filtration after pH adjustment.**

To study the fungal cell pelletization by feeding with NDLH, *M. isabellina* was cultured with three different media: basic medium (with pure xylose), NDLH with or without filtration. As shown in Figure 4A, very loose floccules were observed when *M. isabellina* was cultured with the basic medium, and the whole culture broth showed a high viscosity. The basic medium was not suitable for *M. isabellina* to form pellets although it gave a good biomass and lipid production (Table 1). When *M. isabellina* was cultured with NDLH, the pellets were generated within 24 hours, but the sizes of pellets were different between the treatment methods (Figure 4B and 4C). The average diameters of fungal pellets on NDLH with or without filtration were 0.3 mm and 1.1 mm respectively. In general, the pellet formation can be influenced by many factors, such as inoculum concentration, addition of nuclei or polymer, medium composition, pH, dissolved oxygen and shear forces [34]. Xia et al. [14] reported that the filamentous fungus *Mucor circinelloides* could attach on the surface of calcium carbonate to form pellets. In this study, after the dilute sulfuric acid pretreatment, neutralization with calcium hydroxide led to the production of gypsum, a relatively insoluble coagulant salt which could be served as nuclei for the fungi to develop pellets. Although it took ten minutes for settlement after pH adjustment, some gypsum still remained in NDLH and led to large size pellets generation. The filtration removed most of the solid particles, but some precipitates appeared in the NDLH (without inoculation of fungal spores) after 24 hours, which might be the reason why some small size fungal pellets were produced when culturing with filtered NDLH. 

It was important that the oleaginous fungi formed pellets when fed with NDLH for three reasons: (1) the pelletization could improve the mixing and mass transfer caused by viscosity, and was preferred in the microbial lipid production because of its easier harvesting compared with the traditional centrifugation [14]; (2) the gypsum produced during dilute acid pretreatment of lignocellulosics was considered to negatively affect the downstream ethanol production process, but it could be used as nuclei for the formation of fungal pellets to benefit the lipid production and reduce the cost for the addition of other nuclei or polymer [35]; (3) the fermentation process could be further simplified since the filtration of NDLH was not necessary.

### The perspective of fungal lipid-based biodiesel production from lignocellulosic biomass

The evaluation of SCO and biodiesel yields from lignocellulosic biomass is shown in Table 4. Linde et al. [36] reported that 39.6 g glucose and 21.6 g xylose could be obtained from 100 g wheat straw with dilute sulfuric acid pretreatment followed by enzymatic hydrolysis. Estimations based on the lipid yield of *M. isabellina* in this work (Table 1) suggested that 103 kg lipid could be produced by fungi per ton of wheat straw. It was approximately 29 gallon biodiesel per ton of wheat straw according to Liu and Zhao’ [37] results that 91% recovery rate could be achieved by direct methanolysis of *M. isabellina* biomass. Actually, the yield can be improved since the cultivation has not been optimized yet. The theoretical biodiesel yield is 56 gallons per ton of wheat straw, resulting in 3.19 billion gallons of biodiesel produced each year potentially according to the annual wheat straw supply of 57 million dry tons in the US [18,38]. The Energy Independence and Security Act (EISA) of 2007 mandates the specific yearly target of 36 billion gallons for advanced biofuels [39]. Actually, the annual wheat straw yield is only 4% of the total lignocellulosic biomass yields [18], therefore producing fungal lipid based biodiesel from lignocellulosic biomass exhibits great promise for meeting the national requirement. 

**Table 4:** Estimated biodiesel yield from fungal lipids grown with wheat straw

	**This study**	**Theoretical**
Lipid yield (kg ton^-1^ glucose)	195	320^a^
Lipid yield (kg ton^-1^ xylose)	121	340^a^
Lipid yield (kg ton^-1^ wheat straw)^b^	103	200
Biodiesel yield (gal ton^-1^ glucose)	55	89
Biodiesel yield (gal ton^-1^ xylose)	34	95
Biodiesel yield (gal ton^-1^ wheat straw)^c^	29	56
Current biodiesel yield in US (billion gal y^-1^)^d^	0.32	0.62
Potential biodiesel yield in US (billion gal y^-1^)^d^	1.65	3.19

^a^ Theoretical lipid yields on glucose and xylose are described by Papanikolaou and Aggelis [38].

^b^ 39.6 g glucose and 21.6 g xylose can be obtained from 100 g wheat straw with dilute sulfuric acid pretreatment followed by enzymatic hydrolysis [36].

^c^ The biodiesel yield from direct methanolysis of *M. isabellina* biomass is 91.0% [37].

^d^ The current and potential annual wheat straw yields in US are 11 million dry tons and 57 million dry tons respectively [18].

From the economical assessment aspect, carbon source attributes up to 75% of the total cost for producing biodiesel from SCO [2]. By using commercial raw sugar as feedstock, with an average price at about $852 ton^-1^ (duty fee paid) based on the data of IntercontinentalExchange (ICE, http://www.theice.com) in 2011, the fungal lipid based biodiesel production cost is $20.8 gal^-1^ (based on biodiesel yield on glucose in this study, Table 4). However, 70% of this cost will be cut if lignocellulosic biomass is used as the feedstock. According to the latest estimations released from the National Renewable Energy Laboratory [40], the selling price for lignocellulosic biomass derived sugar is only $257 ton^-1^ (including the costs of feedstock, handling, pretreatment, enzymatic hydrolysis, waste treatment, fixed cost, capital depreciation and the associated tax), which will result in the biodiesel production cost of $6.3 gal^-1^. Based on the theoretical yield (on glucose) in Table 4, the biodiesel manufacturing cost can be potentially reduced to $3.8 gal^-1^ from lignocellulosic biomass, however, there is still a disparity with the US DOE’s target for renewable diesel of $2.8 per gallon by 2017 [41]. Therefore, it is very necessary to make further technical improvements for economical application not only on the fermentation but also on other processes, such as harvesting, extraction, transesterification, high value co-product production, etc.

## Conclusions

This is the first report to investigate the capabilities of filamentous fungi for lipid production with the hydrolysate from dilute sulfuric acid pretreatment of wheat straw. All of the selected oleaginous fungi could grow on the hydrolysates with or without detoxification. Wherein, three fungal strains, including *A. terreus*, *M. isabellina* and *M. vinacea*, showed the highest tolerance to the inhibitors existing in the hydrolysate. The highest lipid content of 39.4% was achieved by *M. isabellina* on NDLH. In addition, the filamentous fungi could form proper pellets to benefit the downstream harvesting process when cultured on NDLH. Overall, cultivation of filamentous oleaginous fungi with lignocellulosic biomass showed great promise for biodiesel production.

## Methods

### Dilute sulfuric acid pretreatment of wheat straw

Wheat straw was obtained from Pullman, WA. The milled wheat straw was mixed with 2% (v/v) dilute sulfuric acid at a solid loading of 10% (w/v) and pretreated in an autoclave at 121°C for 60 min. After cooling, the liquid hydrolysate was separated by centrifugation. Calcium hydroxide was used to adjust the pH to 5.5. After 10-min settling, the supernatant was prepared as NDLH. And then the NDLH was filtered with a 0.22 μm membrane (Millipore, MA) for use as a fermentation substrate.

### Detoxification of the hydrolysate

The detoxification process was similar with that described by Yu et al. [22]. Briefly, the original liquid hydrolysate (without pH adjustment) was heated to 42°C while stirring, and then calcium hydroxide was added to increase the pH to 10.0. The temperature would increase to 50–52°C by addition of calcium hydroxide, and thereafter the mixtures were kept stirring at 50°C for 30 min. After detoxification, the liquid was separated and re-acidified to pH 5.5 with sulfuric acid, followed by passing through a 0.22 μm membrane (Millipore, MA).

### Strains and media

Eleven potential lipid producing fungi were investigated: *A. niger* (NRRL 364), *A. terreus* (NRRL 1960), *C. globosum* (NRRL 1870), *C. elegans* (NRRL 2310), *M. isabellina* (NRRL 1757), *M. vinacea* (ATCC 20034), *M. circinelloides* (NRRL 3628), *N. fischeri* (NRRL 181), *R. oryzae* (NRRL 1526), *M. plumbeus* (CBS 295.63), *T. lanuginosus* (ATCC 76323). All the strains were kept on potato dextrose agar (PDA) at 4°C. The compositions of the basic medium were (g L^-1^): (NH_4_)_2_SO_4_, 0.5; KH_2_PO_4,_ 7.0; Na_2_HPO_4_, 2.0; MgSO_4_·7H_2_O, 1.5; CaCl_2_·2H_2_O, 0.1; FeCl_3_·6H_2_O, 0.008; ZnSO_4_·7H_2_O, 0.001; CuSO_4_·5H_2_O, 0.0001; Co(NO_3_)_2_·H_2_O, 0.0001; MnSO_4_·5H_2_O, 0.0001; yeast extract, 0.5 [42]. Glucose (30 g L^-1^) and xylose (30 g L^-1^) were used as the carbon source respectively. Cultures were conducted in triplicate in 250 mL Erlenmeyer flasks containing 50 mL medium in an orbital shaker at a rotary rate of 200 rpm, and inoculated with 1 ml of spore suspension (1 × 10^7^ spores). The temperature was maintained at 28°C, except *T. lanuginosus* (50°C).

To evaluate the capability of utilizing hydrolysates, the selected lipid producing fungi were cultured in 250 mL Erlenmeyer flasks containing 50 mL each of either NDLH or DLH, as well as 0.4 g L^-1^ MgSO_4_·7H_2_O, 2.0 g L^-1^ KH_2_PO_4_, 0.003 g L^-1^ MnSO_4_·H_2_O, 0.0001 g L^-1^ CuSO_4_·5H_2_O, and 1.5 g L^-1^ yeast extract. The culture conditions were the same as the description above.

### Analyses

The fungal biomass was harvested and washed three times by distilled water, and then freeze-dried to a constant weight. The analysis of fatty acids was performed by Hewlett Packard 5890 gas chromatograph with a Supelco SP-2560 capillary column (100 m × 0.25 mm × 0.20 μm). The conditions for GC were the same as the description by O'Fallon et al. [43]. Tridecanoic acid (C13:0) was used as the internal standard.

Monosugars were analyzed using a Dionex ICS-3000 ion chromatography system equipped with a CarboPac TM PA 20 (4 × 50 mm) analytical column, and CarboPac TM PA 20 (3 × 30 mm) guard column (Dionex Corporation, CA) [22]. Acetic acid, furfural and HMF were determined via High-performance liquid chromatography (HPLC) with a Biorad Aminex HPX-87 H column (Bio-Rad Laboratories, CA) and a refractive index detector as described by Sluiter et al. [44].

### Statistics

The experimental data were statistically analyzed with ANOVA using SAS 9.2 (SAS Institute Inc.). All values were presented as the average of three independent measurements with significance declared at P <0.05.

## Abbreviations

DLH,Detoxified liquid hydrolysate; NDLH,Non-detoxified liquid hydrolysate; SCO,Single cell oil; GLA,γ-linolenic acid; AA,Arachidonic acid; FAME,Fatty acid methyl ester; DCW,Dry cell weight; HMF,Hydroxymethylfurfural; PUFA,Polyunsaturated fatty acid; EISA,Energy Independence and Security Act; ICE,IntercontinentalExchange.

## Competing interests

The authors declare that they have no competing interests.

## Authors’ contributions

YZ participated in the conception, design, data collection and analysis, and drafted the manuscript. XY and JZ assisted the laboratory work, results interpretation and the manuscript revision. SC participated in the critical discussion of the results and revised the manuscript. All authors read and approved the final manuscript.
